# Reply to the Letter from Waddell and Colleagues

**Published:** 1992-12

**Authors:** J. Estèeve


 © Macmillan Press Ltd., 1992

LETTER TO THE EDITOR

### Reply to the Letter from Waddell and Colleagues

Sir – When a case is lost, a good lawyer is needed to try to save the accused. Waddell *et al.* have tried hard to extract the last drop of evidence which could shed some doubt on the association between alcohol drinking and cancer. Unfortunately, the effort is so laboured that it is hardly necessary to refute their arguments.

It is, however, necessary to comment on some of them. The authors state that laryngeal cancer is of special interest because it is a site which does not have direct contact with ingested alcohol; I agree with them but I would reach the opposite conclusion; the fact that those sites with direct contact with alcohol have a greater risk for this exposure is an argument for the causal effect of alcohol. The fact that epilarynx (the border of the larynx) is similar to hypopharynx and not to endolarynx is an argument in the same direction. I agree that in the study where this is shown (Tuyns *et al.*, 1988), the information on non-smokers is weak, but unfortunately it is quoted incompletely: seven cases are observed against 9.4 expected in the 0–40 g category of alcohol drinking, but 15 cases were observed against 12.6 expected in the 40+ category. Due to small numbers this is not significant but the pattern in non-smokers is consistent with a full synergy between alcohol consumption and smoking in causing laryngeal cancer (Estève & Tuyns, 1988). The result in non-smokers is significant for hypopharynx/epilarynx, and there is certainly no J-shape dose-effect relationship for these sites of cancer. Although the risk is formally significant only after an alcohol consumption of more than 40 g/day, the estimate of the dose-response shows a steady increase of risk with dose. It would be necessary to carry out a much larger study to get a reliable estimate at low doses as in any other exposure/disease association. Even for cancer of the endolarynx, the suggestion of a threshold cannot be considered as guaranteed.

Cancer of the oesophagus is probably, as quoted by Waddell *et al.*, the most interesting. It is in effect the site where the synergy with tobacco is the weakest. But when careful attention is paid to the data, it appears that the weak synergy results from the fact that the effect of alcohol in non-smokers and at low levels of smoking is too large to fit perfectly a multiplicative model (Estève & Tuyns, 1988).

In addition to the argument drawn from analytical epidemiology studies, it is of interest to quote those taken from descriptive epidemiology. It has been shown in several countries where alcohol drinking is common that changes in alcohol consumption are shortly followed by consistent and parallel changes in larynx, oesophageal and cirrhosis mortality (Tuyns *et al.* (1988), McMichael (1978), Coleman & Estève (1992)).

All in all, the evidence in humans is so compelling that the discussion is hardly needed. In fact, epidemiologists have reached a consensus which was correctly reflected in IARC Monograph No. 44. It is surprising that other scientists could still cast doubt on the carcinogenicity of alcoholic beverages in humans. Is there a need for more general training in epidemiology and biostatistics?

Dr J. Estève,
Adviser on Biostatics,
IARC,
150 Cours Albert-Thomas,
69372 Lyon Cedex 08, France.

**References**

COLEMAN, M. & ESTÈVE, J. *Time Trends in Cancer Incidence and Mortality*. IARC Scientific Publications No. 121. International Agency for Research on Cancer, Lyon (in press).

ESTÈVE, J. & TUYNS, A.J. (1988). Models for combined action of alcohol and tobacco on risk of cancer: What do we really know from epidemiological studies? In: *Chemical Carcinogenesis*. Feo, F., Pani, P., Columbano, A., Garcea, R. (eds.) Proceedings of the Fourth Sardinian International meeting, 23–27 October 1987, Alghero, Italy. Plenum Publishing Corporation, pp. 649–655.

IARC (1988). *Alcohol Drinking*. IARC Monographs on the Evaluation of Carcinogenic Risks to Humans, IARC Scientific Publications No. 44.

McMICHAEL, A.J. (1978). Increases in laryngeal cancer in Britain and Australia in relation to alcohol and tobacco consumption trends. *Lancet*, **i**, 1244–1247.

TUYNS, A.J., ESTÈVE, J., RAYMOND, L., BERRINO, F., BENHAMOU, E., BLANCHET, F., BOFFETTA, P., CROSIGNANI, P., DEL MORAL, A., LEHMANN, W., MERLETTI, F., PÉQUIGNOT, G., RIBOLI, E., SANCHO-GARNIER, H., TERRACINI, B., ZUBIRI, A. & ZUBIRI, L. (1988). Cancer of the larynx/hypopharynx, tobacco and alcohol. *Int. J. Cancer*, **41**, 483–491.


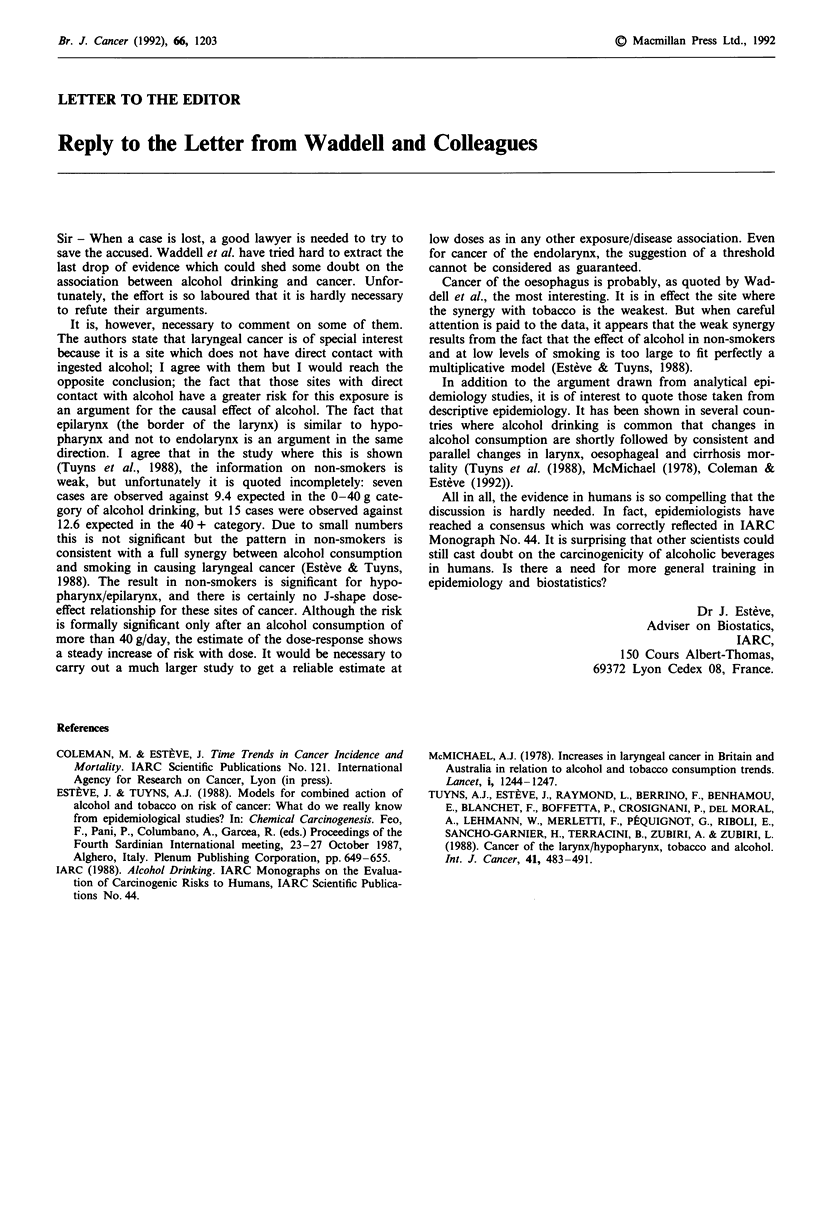


## References

[OCR_00096] McMichael A. J. (1978). Increases in laryngeal cancer in Britain and Australia in relation to alcohol and tobacco consumption trends.. Lancet.

[OCR_00101] Tuyns A. J., Estève J., Raymond L., Berrino F., Benhamou E., Blanchet F., Boffetta P., Crosignani P., del Moral A., Lehmann W. (1988). Cancer of the larynx/hypopharynx, tobacco and alcohol: IARC international case-control study in Turin and Varese (Italy), Zaragoza and Navarra (Spain), Geneva (Switzerland) and Calvados (France).. Int J Cancer.

